# Symptomatic ileocolic pseudoaneurysm following laparoscopic ileocecectomy for Crohn's disease: A case report

**DOI:** 10.1016/j.ijscr.2022.107059

**Published:** 2022-04-08

**Authors:** M. AbuDalu, Y. Munz, G. Ohana

**Affiliations:** Department of General Surgery, Barzilai Medical Center, Ashkelon, Israel

**Keywords:** Case report, Crohn's disease, Pseudoaneurysm, Ileocecectomy, Vascular involvement

## Abstract

**Introduction:**

Crohn's disease is prevalent worldwide. It is an idiopathic, chronic and relapsing disease, characterized by chronic inflammation of any part of the gastrointestinal tract. Vascular involvement rarely occurs in Crohn's patients. However, the chronic inflammatory process leads to structural and functional changes in the vascular endothelium. We present a case of ileocolic artery pseudoaneurysm after laparoscopic ileocecectomy in a Crohn's patient.

**Case presentation:**

We report a case of a 26-years-old male diagnosed with Crohn's disease 4 months prior to an elective laparoscopic ileocecectomy. Before the operation, the patient suffered from severe terminal ileitis and typhlitis with signs of micro perforation. 3 weeks following his discharge, the patient arrived at the ER complaining of severe right lower abdominal pain. Computerized Tomography (CT) scans revealed a 35 mm ileocolic pseudoaneurysm that was treated urgently with coil-embolization via angiography.

**Discussion:**

Until recently, few reports regarding the involvement of pseudoaneurysm of mesenteric arteries in relation to bowel resection surgeries have been reported. It is believed that the chronic inflammatory process induces a potent effect on the vascular endothelium, leading to thick, inflamed and highly friable mesenteric vessel walls. We propose that the pseudoaneurysm occurred as a result of surgical intervention on a thickened and diseased mesentery artery branch.

**Conclusion:**

Inclusion of mesenteric resection in Crohn patients' undergoing bowel resection may be an innovative way to avoid this complication and to reduce recurrence.

## Highlights

• Crohn’s disease is characterized by chronic inflammation of any part of the gastrointestinal tract.
• Mesenteric vascular involvement in Crohn’s’ disease.
• A case of ileocolic artery pseudoaneurysm after laparoscopic ileocecectomy in a Crohn’s patient.
• Mesenteric resection in Crohn patients’ undergoing bowel resection may be an innovative way to reduce recurrence.

## 1 Introduction

The annual incidence of Crohn's disease (CD) worldwide is ranging from 3 to 20 cases per 100,000 [1]. It is a transmural granulomatous [2] inflammatory bowel disease that is characterized by chronic inflammation of any part of the gastrointestinal tract. Although the cause of CD is still unclear, several factors have been implicated, including a dysregulated immune system, an altered microbiota, genetic susceptibility, and environmental factors [3].

Crohn's disease is characterized by macroscopic changes of the mesentery including thickening, stiffness and hypertrophy of mesenterial fat (creeping fat) [4]. Microscopically, mesenteric adipose tissue in Crohn's patients demonstrate fibrosis perivascular inflammation, infiltration of stromal cells, thickened lymphatic vessels and perineuronal chronic inflammation [5].

Regardless of the recent advances in medical therapy for CD, especially with the development of biological therapy, surgical treatment with intestinal resection still plays an important part in the management of these patients [6]. Although open resection is still used by many surgeons in CD treatment, the laparoscopic approach is being used with increasing frequency [7]. However, laparoscopic surgery in CD has been shown to be challenging due to the inflammatory and recurrent characteristics of this condition. For example, the active inflammation, mesentery infiltration and friability, presence of fistulae, adhesions and concomitant immunosuppression often complicate the laparoscopic procedure [6]. Common complications after this type of operation include wound infection, intra-abdominal collection and rarely, anastomotic leaks. Here, we present a case of ileocolic artery pseudoaneurysm after laparoscopic ileocecectomy in a Crohn's patient.

## 2 Case presentation

**Fig. 1:**
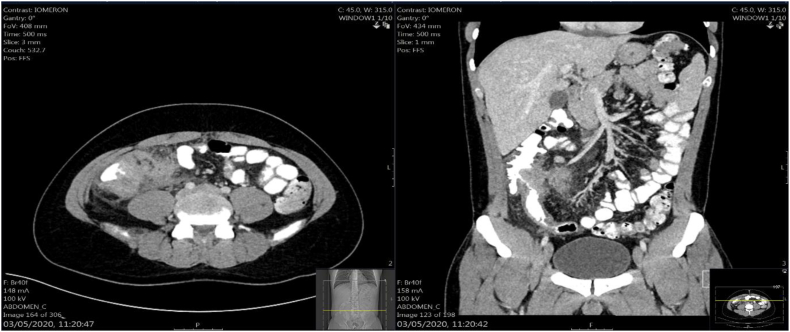
Abdominal CT scan showing signs of terminal ileitis and typhlitis with micro perforation.

A 26 years-old male with a 4-month history of Crohn's disease, who was treated only with antibiotics with no signs of improvement, arrived at the emergency department complaining from severe episodes of abdominal pain. Upon his arrival to the emergency department, physical examination revealed right lower quadrant tenderness and palpated infiltration with signs of local peritonitis. Laboratory results showed signs of inflammation with leukocytes count of 10.7k and a CRP of 54. CT scan revealed signs of severe inflammatory process involving the terminal ilium and the cecum with signs of micro perforation (Fig. 1). The patient had no history of any other debilitating conditions and no relevant family history.

**Fig. 2:**
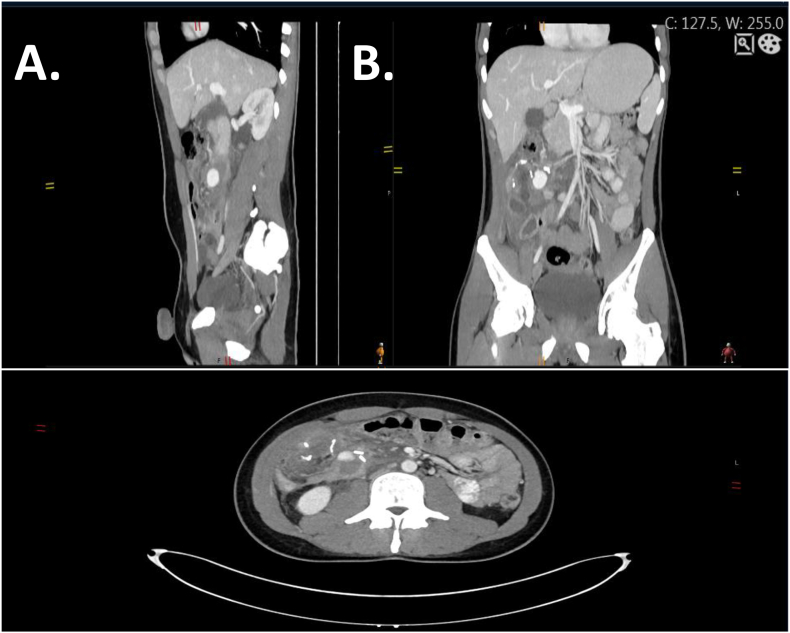
CTA scan showing ileocolic pseudoaneurysm. A. Sagittal view. B. Coronal view. C. Axial view.

**Fig. 3:**
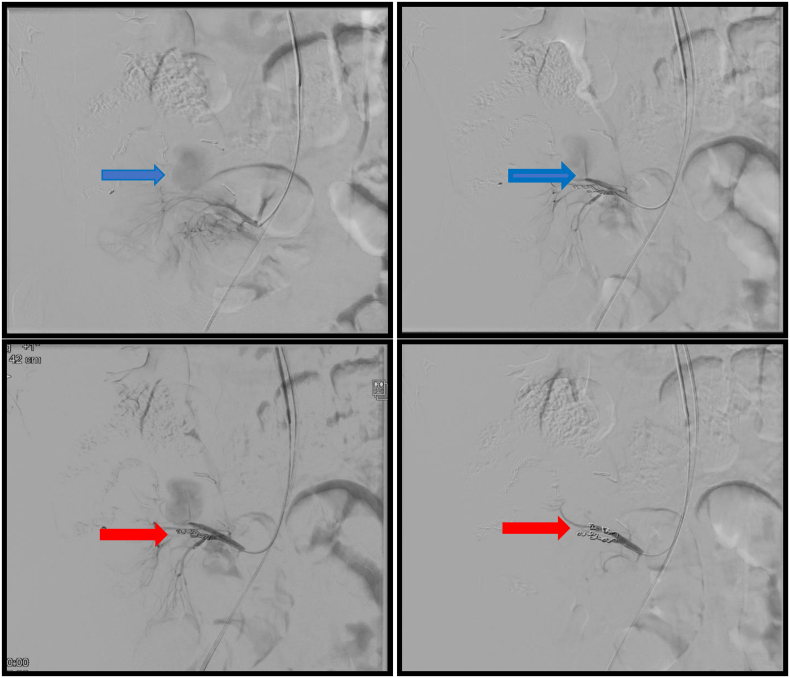
Angiography showing the site of the pseudoaneurysm (blue arrow) and cord embolization (red arrow). (For interpretation of the references to colour in this figure legend, the reader is referred to the web version of this article.)

**Fig. 4:**
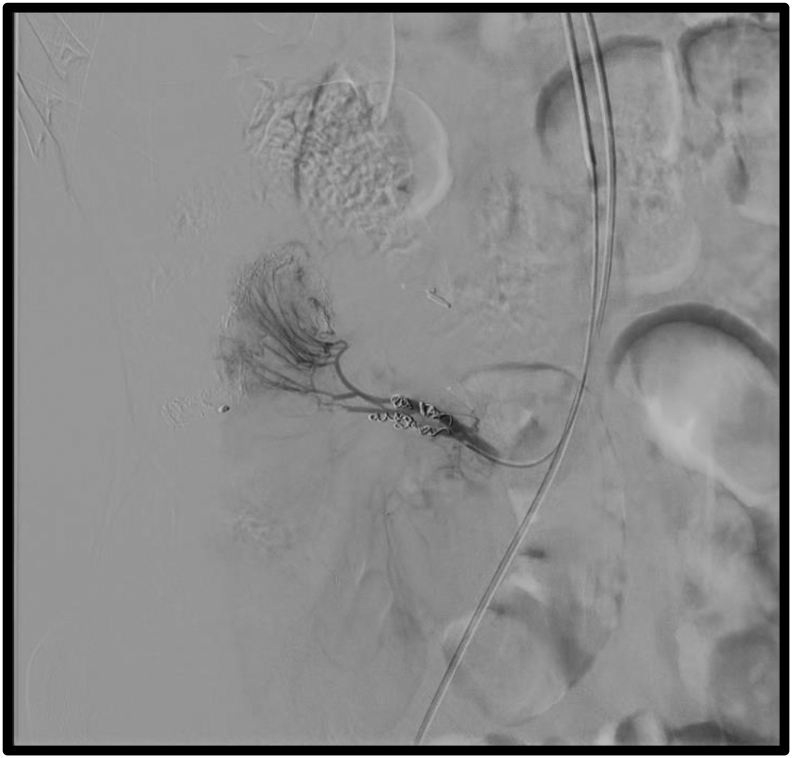
Angiography showing the final result of the angioembolisation procedure.

After a multidisciplinary discussion on this case, a laparoscopic ileocecectomy was proposed which was performed in the General Surgery Department by two highly expertise senior surgeons. The surgery was uneventful, and the patient was discharged a week after. Three weeks following his discharge, the patient arrived at the emergency department suffering from a worsening persistent abdominal pain. No fever was detected. Blood tests revealed no signs of inflammation or other serious findings. The patient was subjected to an intravenous contrast enhanced abdominal CT scan. The CT scan revealed a 35 mm ileocolic pseudoaneurysm (Fig. 2). To further access the previous finding of the abdominal CT, the patient underwent an angiography (Fig. 3- upper raw) which revealed a pseudoaneurysm of the stump of the ileocolic artery. The pseudoaneurysm was urgently treated with selective coil-embolization (Fig. 3- lower raw). The procedure was reported to be successful with a complete occlusion of the pseudoaneurysm (Fig. 4). Ten days later, he was scheduled for a follow up. Physical examination was normal, and he was planned for azathioprine (Imuran) therapy. 11 months after the surgery the patients returned for a second follow up. No signs of further complications were noted. The Patient was satisfied with treatment and outcome. It is noteworthy to mention that the work has been reported in line with the SCARE 2020 criteria [8].

## 3 Discussion

The laparoscopic approach to CD represents the most important advancement in surgical treatment for the management of CD [6]. Laparoscopic ileocolic resection is currently the most commonly performed operation especially in primary CD [9]. Furthermore, in recurrent disease or CD complicated by fistulae and abscesses, when performed by experienced surgeons, ileocolic resections by laparoscopic approach seems to be safe and feasible, without increasing the rates of postoperative complications [10].

To date, pseudoaneurysm of mesenteric arteries as a complication of bowel resection has been rarely reported in the literature. In our case, we propose that the pseudoaneurysm occurred due to the surgical intervention carried out on thickened and diseased mesenteric artery branch. This phenomenon has been reported before by Edden et al. [11] and Salinas et al. [12]. They postulated that a branch of the ileocolic artery that was not adequately ligated due to the thick and inflamed mesentery had bled and formed the pseudoaneurysm encapsulated by mesenteric fat.

There is emerging evidence suggesting that the mesenteric structures, including nerves, blood vessels, lymphatics, fat, and connective tissues, play a crucial role in the pathogenesis and disease progression of CD, that is, the dysregulation and “cross talk” between those components that contribute to chronic intestinal inflammation [5]. Due to the crucial role of the mesentery in Crohn's disease disposition, we suggest that the inclusion of mesenteric resection in Crohn patients' undergoing bowel resection may be an innovative way to avoid this complication and to reduce recurrence. Coffey et al. [13] demonstrated that adoption of mesenteric based strategies is associated with improved clinical outcomes after ileocolic resection in Crohn's disease. Mesenchymal inputs contribute to mesenteric abnormalities and their reduction may partly explain the benefits of mesenteric-based surgical strategies.

## 4 Conclusion

This is the third case of mesenteric pseudoaneurysm following bowel resection for Crohn's disease to our knowledge. It may be due to the inflamed mesenteric nature of Crohn's patients that might predispose them to developing these pseudoaneurysm. Inclusion of mesenteric resection in Crohn patients' undergoing bowel resection may be a way to avoid this complication.

## Funding

No funding.

## Ethical approval

The patient has given his informed consent for this publication.

## Consent

Written informed consent was obtained from the patient for publication of this case report and accompanying images. A copy of the written consent is available for review by the Editor-in-Chief of this journal on request.

## Author contribution

A.M wrote the paper. M.Y. and O.G. revised the manuscript critically and provided academic guidance. All authors have read and agreed to the published version of the manuscript.

## Provenance and peer review

Not commissioned, externally peer-reviewed.

## Research registration

Not applicable.

## Guarantor

Dr. Ohana Gil.

Dr. Munz Yaron.

## Declaration of competing interest

No conflict of interest.
